# Use of the iliac-outlet and iliac-inlet combined views in percutaneous posterior column retrograde screw fixation

**DOI:** 10.1007/s00402-023-04939-2

**Published:** 2023-06-07

**Authors:** Stefano Cattaneo, Claudio Galante, Elena Biancardi, Marco Domenicucci, Marco Paderno, Antonio Pianelli, Giuseppe Milano, Alessandro Casiraghi

**Affiliations:** 1 Department of Bone and Joint Surgery, ASST Spedali Civili, Piazzale Spedali Civili 1, 25123 Brescia, BS Italy; 2 Department of Medical and Surgical Specialties, Radiological Sciences, and Public Health, University of Brescia, Viale Europa 11, 25123 Brescia, BS Italy

**Keywords:** Posterior column, Fluoroscopic view, Percutaneous screw, Acetabular fracture, Percutaneous acetabular surgery

## Abstract

Posterior column fractures are common acetabular injuries. Although displaced fractures require open reduction and fixation, undisplaced patterns may benefit from percutaneous screw fixation. The combination of iliac oblique with inlet and outlet views offers an intuitive and panoramic rendering of the bony corridor into the posterior column; lateral cross table view completes the sequence of fluoroscopic projections. Herein we describe the use of outlet/inlet iliac views and a detailed procedure for percutaneous retrograde posterior column screw fixation.

## Introduction

Open anatomical reduction and internal fixation is the standard of care for displaced fracture of the acetabulum [1]. Percutaneous screw fixation is related to satisfactory results in minimally displaced, non-comminuted acetabular fractures, particularly in patients with severe soft tissue injury and increased risk for major surgery [2–6].

The first description of the percutaneous technique advocated the use of supine decubitus and iliac/obturator and inlet/outlet views [3]; however, surgical steps of the procedure are not clearly described. Several studies have investigated acetabular bone corridors morphology and their anatomical variability [7–9]. The relationship between the ischial entry point and the sciatic nerve have been assessed [10]. Navigated and computer-assisted procedures have been developed and compared to traditional fluoroscopic guided techniques [11, 12]. A fluoroscopic-guided technique, performed in prone position, has been described into details, using obturator oblique, iliac oblique and outlet-obturator views [5]. However, supine decubitus is preferred for anaesthesiologic reasons and for easier combination with anterior components fixation.

Percutaneous retrograde posterior column screw fixation remains a challenging procedure and a clear consensus on which fluoroscopic views are most accurate and intuitive is still lacking.

Aim of this study is to present a detailed technique to perform percutaneous retrograde posterior column screw fixation in supine position, using a sequence of inlet and outlet -iliac and cross-table lateral views to assess the bony corridor inside the posterior column.

## Methods

The patient is placed in a supine position on a flat radiolucent table. The ipsilateral lower limb is prepped free in a sterile fashion, in addition to the entire pelvic region, with special attention to the perineal area: a U-shaped drape should be positioned medial to the ischial tuberosity and lateral to the perineum. The draping should be reinforced with adhesive polyethylene sterile band to assure asepsis and stability of draping during hip hyperflexion. The image intensifier is placed contralateral to the injured side. The hip is flexed 90° and slightly externally rotated, to decrease tension on the sciatic nerve. Slight hip hyperflection can be performed to reduce pelvic inclination and facilitate guide wire positioning.

The ischial tuberosity is palpated, and a 10-mm incision is performed with a blade.

**Fig. 1:**
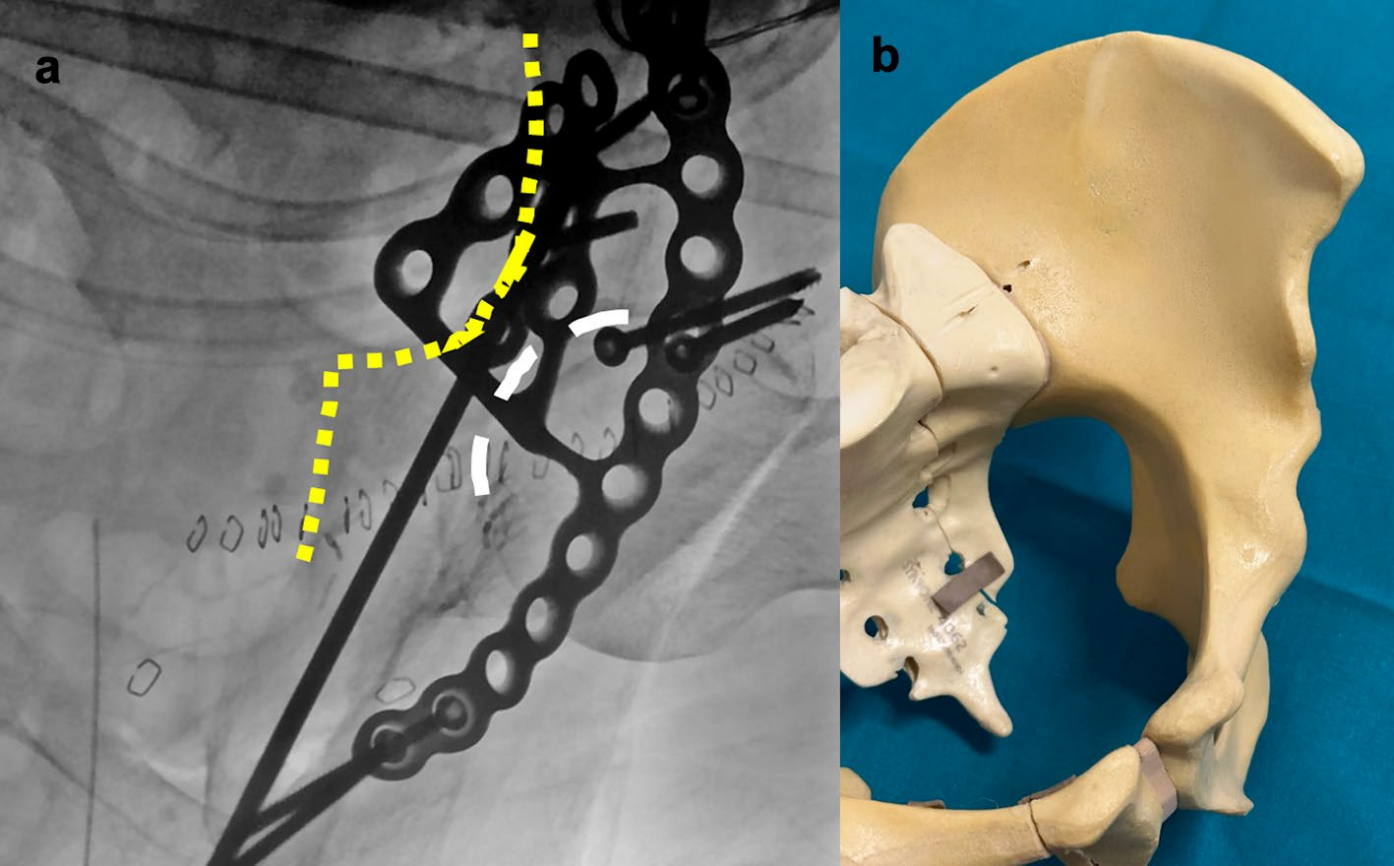
Iliac-outlet view. **a** Fluoroscopic view shows ischial outline (black interrupted line) and guide wire entry point (arrow); **b** sawbone representation of fluoroscopic view

An outlet-iliac oblique view is obtained to assess the correct entry point and the corridor along the midline axis of the ischium. A 3.2 mm guide wire is inserted into the ischium and advanced few millimeters (Fig. 1).

**Fig. 2:**
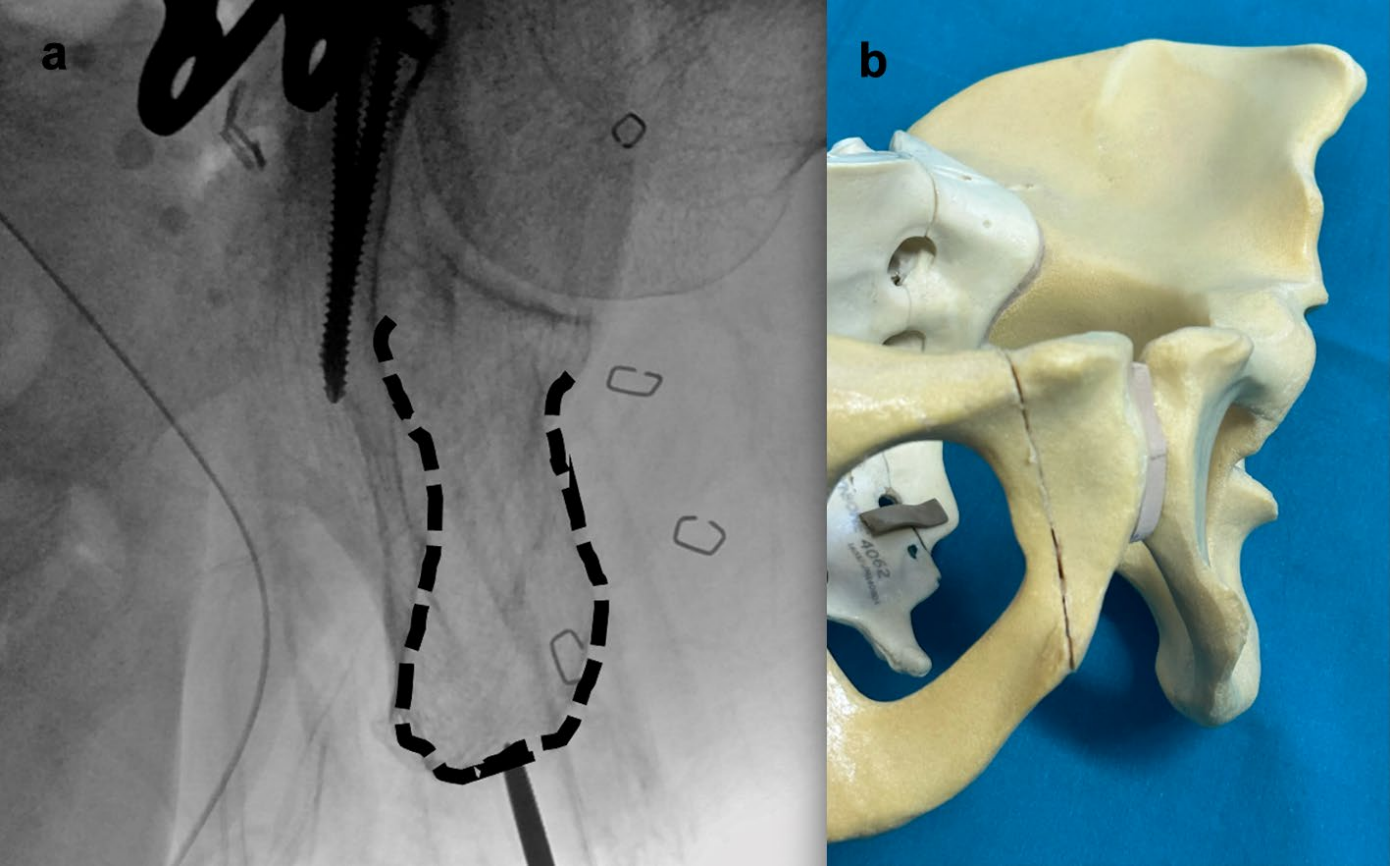
Iliac-inlet view. **a** fluoroscopic view shows posterior column landmarks, ischial spine and sciatic notch (yellow dotted line) and acetabular cavity (white interrupted line). **b** Sawbone rendering of fluoroscopic view

**Fig. 3:**
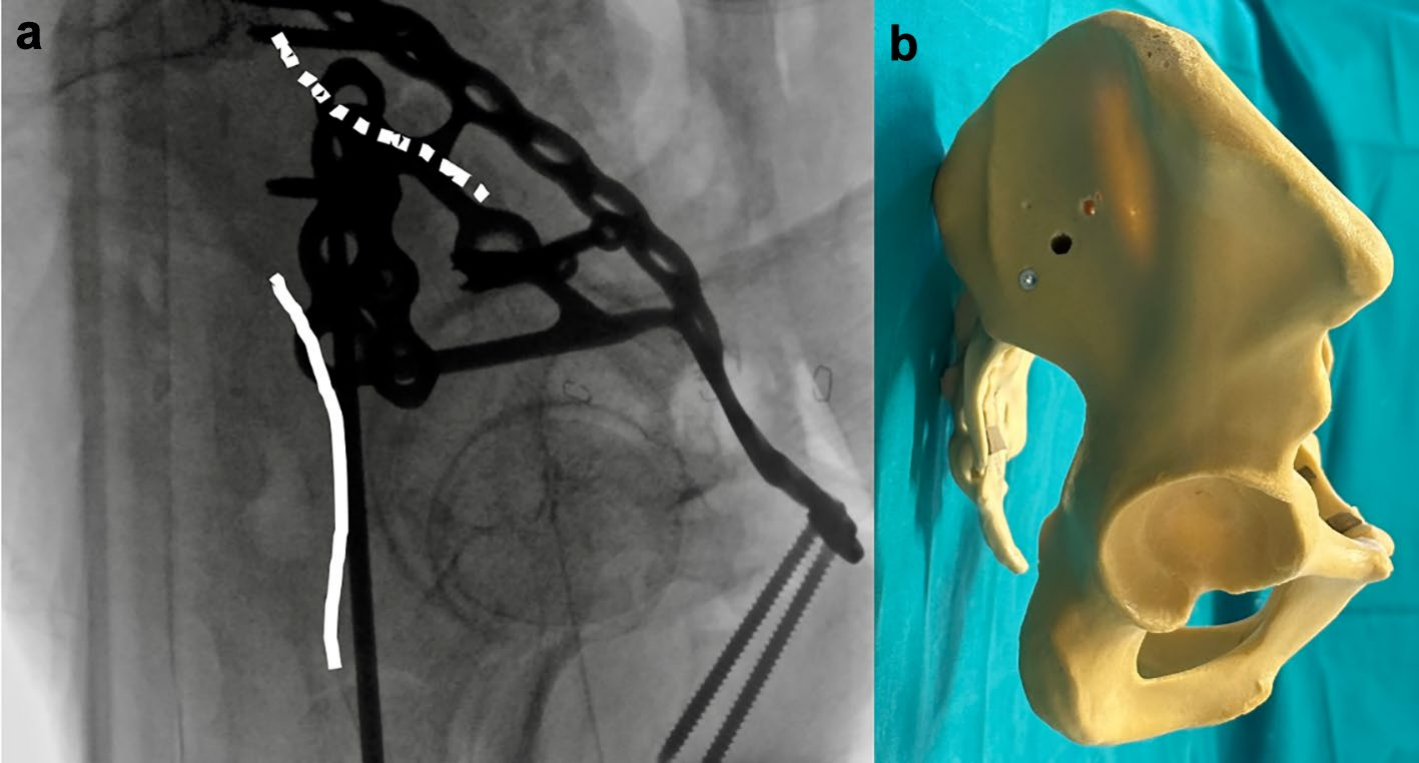
Cross-table lateral view. **a** Fluoroscopic view shows profile of subcotyloid notch and retroacetabular surface (white solid line) and pelvic brim (white dotted line). **b** Sawbone rendering of fluoroscopic view

An inlet-iliac oblique view is obtained to assess the corridor medial to the acetabular cavity and lateral to the greater sciatic notch. (Fig. 2). A cross table lateral view is obtained to assess guide wire position on the sagittal plane, to avoid penetration of the subcotyloid notch or retroacetabular surface (Fig. 3).

Outlet-iliac, inlet-iliac and cross table lateral views are sequentially obtained during guide wire insertion to assess its correct placement. Once the inner cortex of the iliac wing is reached, screw length is obtained with a dedicated measure. A cannulated 6.5-mm drill is used to open the ischial cortex and 8-mm partially threaded cannulated screw (Asnis III Cannulated Screw System, Stryker Corp. Kalamazoo, MI, USA) is implanted, under fluoroscopic control. The skin is closed with 2–0 nylon suture.

Postoperative radiograms are obtained to assess the correct position of the implant and joint congruity.

## Results

Five patients underwent percutaneous retrograde posterior column screw fixation at our Institution (Table 1). Two patients had minimally displaced (< 1 mm) acetabular fractures (1 transverse, 1 anterior column + posterior hemi-transverse) and were treated with a completely percutaneous procedure. One patient had a combined pelvic and acetabulum injury and was treated with open reduction and fixation of the sacral injury, percutaneous retrograde right pubic ramus screw and percutaneous retrograde posterior column screw. The fourth patient had an anterior column posterior hemi-transverse (ACPHT) fracture and was treated with open reduction and plate fixation of the anterior column through an anterior intrapelvic approach; the anterior surgical step resulted in indirect reduction of the posterior column and fixation was achieved with a percutaneous retrograde screw. The fifth patient had a pelvic ring injury with an interruption of the low posterior column.

**Table 1:** Patients Treated with Percutaneous Posterior Column Screw: Demographics and Complications (*N* = 5)

Age (mean)	43,8
Sex, *n* (%)	
Male	2 (40)
Female	3 (60)
Mechanism of injury, *n* (%)	
MVC	2
MCC	1
CC	1
FH	1
Delay to surgery, *d*	9,2
Acetabular fracture pattern, *n* (%)	
Transverse	1 (20)
Anterior column-post hemitransverse	2 (40)
Posterior column	2 (40)
Associated pelvic ring disruption, n (%)	2 (40)
Complications, *n* (%)	
Screw migration	1 (20)
Pre-arthritic changes	1 (20)

*MVC* motor vehicle collision, *MCC* motorcycle collision, *CC* cycle collision, *FH* fell from height

All reassumed full weight-bearing within 10 weeks. One patient had a migration of the screw that unscrew and protruded, impairing sitting. The screw was repositioned.

Postoperative radiographs and computed tomography (CT) scans showed correct screw placement in the posterior acetabular column. Follow-up imaging at 12 months showed fracture healing. None of the patients had neurological impairment. One of the patients showed initial pre-arthritic changes consisting in new femoral neck cam deformity.

## Discussion

Percutaneous acetabular screw fixation has been described as an effective procedure to treat minimally displaced acetabular fractures. Posterior column retrograde fixation can be performed in conjunction with other percutaneous screw positioning or associated to open approaches, to limit the surgical exposure to one open approach. This is particularly relevant in ACPHT fractures, when reduction of the posterior column is performed through an anterior approach.

The bony corridor geometry and variability have been assessed in anatomical and imaging studies. Relationship with neurovascular structures have been investigated.

However, percutaneous posterior column retrograde screw fixation remains a challenging procedure, and there is no clear consensus on which fluoroscopic views are more useful.

Starr et al. and Moushine et al. [2, 4] performed the procedure in supine decubitus with iliac/obturator and inlet/outlet view. A detailed description of fluoroscopic landmarks and a sequence of views is lacking in their studies. Wright Jr. et al. [5] suggested to use obturator oblique, iliac oblique and outlet-obturator views in prone position. The sequential use of different views and their function is elucidated into details. Although the authors outlined the advantages of prone positioning, supine positioning is generally preferable, as it allows easier anesthesiologic management and a combination of percutaneous fixation with anterior open approaches. Recently, a technique using anteroposterior and lateral view to implant antegrade column screw was described, outlining the importance of preoperative planning and lateral assessment of the bony corridor [13].

Navigated and computer-assisted techniques seem to be promising, albeit they need dedicated instrumentation and equipment. Moreover, they are expensive, time consuming and not widely available.

The use of iliac outlet and iliac inlet views offers several advantages. Iliac outlet is a ‘true’ anteroposterior view of the low posterior column and a frontal projection of the ischium triangular section. Therefore, it allows a clear identification of the entry point at the centre of the corridor and detection of its medial and lateral borders. Iliac inlet offers a panoramic view of the posterior column and its relationship with the acetabular cavity. Superimposition of pubis and pubic rami is cleared by the inlet combination. Lateral cross-table view can detect guide pin migration into the sub cotyloid groove or a posterior end point.

Combination of inlet and outlet views to iliac oblique results in clear and intuitive fluoroscopic views.

Two patients had complications. In one case the screw migrated and was substituted. We explained the unscrewing with insufficient purchase in the iliac cortex. One patient showed a new cam deformity of the femoral neck at final follow up. We considered it a consequence of fair reduction of the posterior column fracture line. In none of the cases the complications can be related to the chosen fluoroscopic views. Our study has several limitations: the combination of fluoroscopic views was used on a limited number of patients, with a heterogeneous spectrum of pelvic and acetabular lesions. The procedure appears to be safe and effective. Nonetheless, further studies are needed to determine diagnostic accuracy of iliac-inlet/outlet views.

## Data Availability

All data regarding the reported cases, including preoperative, intraoperative and follow up imaging are available upon request.
